# Gouty tophus on the eyelid

**DOI:** 10.22336/rjo.2026.43

**Published:** 2026

**Authors:** Benyamin Leon Anavi, Nina Staneva Stoyanova

**Affiliations:** 1 Department of Clinical Pathology, “Thorax” Hospital, Plovdiv, Bulgaria; 2 “Luxor” Eye Hospital, Plovdiv, Bulgaria; 3 Department of Ophthalmology, Faculty of Medicine, Medical University of Plovdiv, Bulgaria

**Keywords:** eyelid, gout, tophus

## Abstract

A gouty tophus on the eyelid is a casuistic flash in the routine biopsy flow. There has been a 12th case reported in the literature, along with an analysis of the distribution by age, sex, location, size, and symptoms. Male gender predominates (10:2) within a wide age range (26-79 years), after a several-year history of gout, affecting mainly the canthi and, in only 4 cases, the middle area of the eyelids (the upper eyelid more often), with dimensions ranging from 4 to 14 mm. The tophi can be multiple (in 2 cases). In addition to aesthetic concerns and discomfort, the tophi caused mechanical ptosis of the eyelid on two occasions. A histologically confirmed eyelid tophus may serve as an early diagnostic stigma of the disease, even in the absence of hyperuricemia. Their occurrence may be either a manifestation of disseminated skin tophi or a localized disturbance of the protective alkaline homeostasis of the eyelid tissue.

## Introduction

Gout is a metabolic disease characterized by elevated blood uric acid levels, leading to the deposition of monosodium urate crystals and an inflammatory reaction in and around the joints. The disease presents as acute or chronic arthritis, with accumulation of tophi (Latin “tophus” – stone) outside the joints over the years, and as gouty nephropathy. A tophus is pathognomonic for gout: a usually painless swelling, histologically characterized as a granuloma with proliferation of histiocytes and “foreign body” giant cells surrounding deposits of urate crystals.

A gouty tophus on the eyelid is a casuistic flash in the routine biopsy flow. With only 11 such cases reported so far, gout “winks” at us, pointing to the eyelid as another possible site of manifestation.

## Case report

**Fig. 1:**
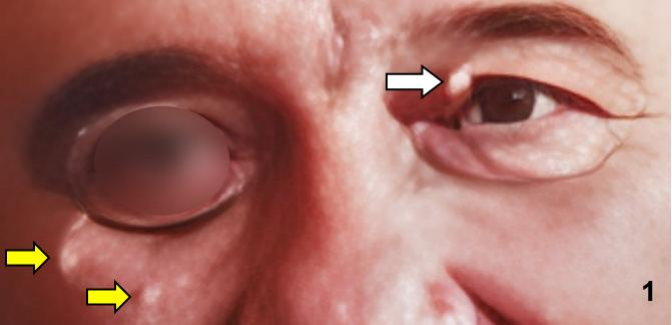
Prominent firm white “lump” (7x5 mm) on the skin in the middle part of the left eyelid (white arrow) and small white nodules on the skin of the right cheekbone (yellow arrows)

A 58-year-old man was admitted to the Ophthalmology Department with a 1.5-year-old “lump” on the medial surface of the upper left eyelid, which had grown to 7-8 mm and had begun to bother him (Fig. 1). The visual acuity of both eyes was 1.0, the intraocular pressure being 15 mm Hg in the right eye and 16 mm Hg in the left eye, without any pathological changes in the anterior and posterior segments of both eyes.

**Fig. 2:**
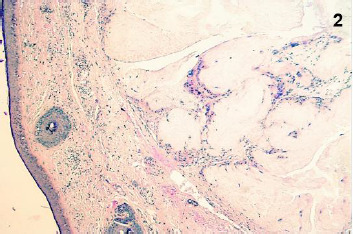
Dermal gouty tophus. Hematoxylin - Eosin, x 100

The excised material (7×5×4 mm) was fixed in buffered formalin. The histological examination, performed with Hematoxylin and Eosin staining, in the dermis, revealed well-defined, rounded basophilic deposits, with slight striations, “bordered” by histiocytes and single multinucleated cells (Fig. 2). The findings were interpreted as a “gouty tophus”.

**Fig. 3:**
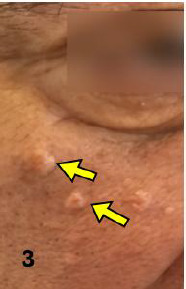
Raised whitish lesions on the right cheekbone (yellow arrows)

**Fig. 4:**
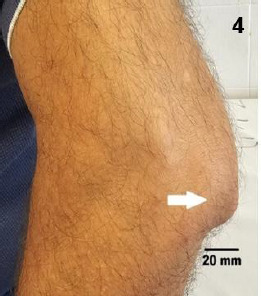
Gouty tophus on the left elbow joint (white arrow)

It turned out that the patient had been suffering from gout for 15 years, with repeated arthritis flares affecting the metatarsal, ankle, and knee joints, with moderately difficult movements. He was treated with Colchicine and also with NSAIDs during acute flares. In addition to the removed swelling of the eyelid, on the skin of the face (right cheekbone and left nasolabial fold), there were whitish prominences limited to 5-6 mm (Fig. 1, 3). The patient refused to have them biopsied. On the left elbow joint, there was a protruding painless nodule (20 mm), assessed as a gouty tophus (Fig. 4).

The complete blood count was within normal limits; uric acid levels were elevated – 555 μmol/l, total cholesterol – 6.85 mmol/l, and LDL cholesterol – 5.36 mmol/l; creatinine and urea were within normal limits; urinalysis showed a pH of 6.0.

The patient suffered from arterial hypertension. He did not report diabetes mellitus, nephrolithiasis, or alcohol abuse.

## Discussion

It is estimated that, as of 2020, 56 million people globally are affected by gout, with projections indicating an increase to 96 million by 2050 [1]. Gout most often affects middle-aged men and, less often, postmenopausal women. The rarity of ocular changes in gout contrasts with the disease’s frequency, yet these complications are highly variable. Urate deposits are reported in every structure of the eye: the conjunctiva, cornea, iris, sclera, and orbit, as well as in association with dry eye syndrome, red eye, uveitis, elevated intraocular pressure, and cataracts. In most cases, the patients have chronic gout, but some present with only ocular symptoms and no other manifestations [2]. This makes rare eyelid tophi particularly interesting, as they may be the first clinical stigmata of undiagnosed gout.

A total of 11 cases of gouty tophi on the eyelids have been published in addition to the present case (Table 1) [3-13]. As expected, males predominate (10:2), with ages ranging from 26 to 79 years. The duration of gout before the appearance of the eyelid tophus exceeds 3 years (up to 20 years). Most patients have experienced multiple gout attacks, with different localizations of arthritis and periarticular tophi. However, the eyelid lesion may also be an early manifestation of the disease [1,3,10], even in the absence of hyperuricemia [7,10].

**Table 1:** Cases of gouty tophi on the eyelids

Author *… et al*.		Publ.	Age	Sex	Gout	Lesion	Tophus	Size (mm)	Other lesions
					Duration (years)				
1	De Monteynard [3]	1986	62	F	-	2 days?	Lateral canthus	-	-
2	Morris [4]	2003	44	M	-	1	Lateral canthus	6×5×4	-
3	Yen [5]	2005	27	M	3	3 months	Medial canthus	11×5×5	1^st^ metatarsal
4	Jordan [6]	2008	68	M	20	2	Medial canthus	5×6×4	Elbow
5	Yang [7]	2008	64	M	-	9	Middle upper lid	14×10×8	*Mechanical ptosis*, elbows, feet hands
6	Nakatsuka [8]	2016	41	M	10	1	Lateral canthus	4×7×4	Ankle +1^st^ metatarsal
7	Sing-Hui [9]	2017	79	M	Normal uric acid	1	Middle upper lid	9×24×14	*Complete mechanical ptosis*
8	Philteos [10]	2019	41	M	3	1	Temporal upper lid	4×5×4	Knee
9	Mikkilineni [11]	2020	37	F	?	7 months	Bilateral cantuses, upper lid	6 × 5 3 × 3 2 × 1	-
10	Meyer [12]	2024	51	M	No history of gout	-	Bilateral lower lids	?	-
11	Lv [13]	2025	26	M	4	-	Lateral canthus	2 lesions 4×3 6×5	-
12	Our case	2025	58	M	15	1,5	Medial upper lid	7×5×4	Elbow

Lesions in the eyelid canthi predominate (7 observations). In four instances (including the present case), the skin of the upper eyelid was affected. However, multiple tophi on the eyelids have been reported as well [9,11], and only once on both lower eyelids [10]. A central depression with superficial crusting has been described [6].

It can be assumed that the actual frequency of tophi is higher than the registered count. They are painless and occur outside of gout attacks. Because they are small, they are probably neglected and overlooked. Tophi can reach significant sizes (9×24×14 mm), causing not only aesthetic problems and discomfort but also mechanical ptosis of the eyelid [5,7]. In the case of complete ptosis, however, the patient is oncologically ill, undergoing chemotherapy, so that a secondary gout may be assumed [7].

Genetic factors can influence uric acid production and excretion, potentially predisposing individuals to hyperuricemia and tophi, but no familial history was reported in the cases reviewed. No recurrence has been described after removal of an eyelid tophus.

Typical of gouty tophi is their formation in poorly vascularized tissues (auricles, olecranon, and prepatellar bursae, as well as tendons), whereas the eyelids are highly vascularized. Two mutually exclusive hypotheses regarding the genesis of these urate granulomas have been outlined:

1. In our case, we assumed a combination with urate deposits on the skin of the face (Fig. 3, without biopsy examination), i.e., the eyelid lesion may be a manifestation of disseminated skin tophi [14].
2. On the other hand, the rarity of gouty granulomas in the eyelid skin may be due to local biochemical and biophysical features of the tissue. Here, the pH is higher than that of the surrounding facial skin. The good blood supply, relative alkalinity, reduced tissue lipid content, and increased hydration capacity [15] are likely protective factors against urate deposition in the eyelids. If so, the rare eyelid tophi may arise from local disturbance of the tissue barrier in the context of chronic hyperuricemia.

The macroscopic differential diagnosis encompasses the full spectrum of non-invasive eyelid lumps that warrant surgical removal. Fixation of the biopsy material in alcohol is preferable, since the aqueous solution of formalin dissolves urate deposits. The histological picture is pathognomonic. Not every “lump” on the eyelid in a gout patient is due to the underlying disease. An example of this is a published case where histology revealed an epidermal cyst, but it was incorrectly interpreted as a “tophus” [16].

Physicians “see what they know”: each newly described case moves the gouty tophi on the eyelid further away from the realm of casuistry and brings us closer to real knowledge.

## Conclusion

We reported the 12th case in the literature of a gouty tophus on the eyelid, accompanied by an analysis of observations on age, gender, location, size, and symptoms. These findings are too rare to be included in the disease’s diagnostic criteria. Yet they may be the first stigmata of the disease, as they can occur even in the absence of or before hyperuricemia. The macroscopic differential diagnosis of urate granulomas includes the full spectrum of non-invasive lumps of the eyelid skin, whose growth indicates surgical removal. Beyond cosmetic concerns and discomfort, they may also cause eyelid ptosis. The gold standard for diagnosing tophi is histological examination.
